# Prevalence of Splanchnic Vein Thrombosis in Pancreatitis: A Systematic Review and Meta-Analysis of Observational Studies

**DOI:** 10.1155/2015/245460

**Published:** 2015-09-14

**Authors:** Wenda Xu, Xingshun Qi, Jiang Chen, Chunping Su, Xiaozhong Guo

**Affiliations:** ^1^The 251st Hospital of PLA, Zhangjiakou 075000, China; ^2^Department of Gastroenterology, Shenyang General Hospital of PLA, Shenyang 110840, China; ^3^Library of Fourth Military Medical University, Xi'an 710032, China

## Abstract

Splanchnic vein thrombosis (SVT) may be negatively associated with the prognosis of pancreatitis. We performed a systematic review and meta-analysis of literatures to explore the prevalence of SVT in pancreatitis. All observational studies regarding the prevalence of SVT in pancreatitis were identified via PubMed and EMBASE databases. The prevalence of SVT was pooled in the total of patients with pancreatitis. And it was also pooled in the subgroup analyses according to the stage and causes of pancreatitis, location of SVT, and regions where the studies were performed. After the review of 714 studies, 44 studies fulfilled the inclusion criteria. Meta-analyses showed a pooled prevalence of SVT of 13.6% in pancreatitis. According to the stage of pancreatitis, the pooled prevalence of SVT was 16.6% and 11.6% in patients with acute and chronic pancreatitis, respectively. According to the causes of pancreatitis, the pooled prevalence of SVT was 12.2% and 14.6% in patients with hereditary and autoimmune pancreatitis. According to the location of SVT, the pooled prevalence of portal vein, splenic vein, and mesenteric vein thrombosis was 6.2%, 11.2%, and 2.7% in pancreatitis. The prevalence of SVT in pancreatitis was 16.9%, 11.5%, and 8.5% in Europe, America, and Asia, respectively.

## 1. Introduction

Splanchnic vein thrombosis (SVT) is one such vascular complication of pancreatitis. SVT involves the portal vein (PV), splenic vein (SplV), and mesenteric vein (MV) with the occurrence in combination or separately [1]. The consequence of pancreatitis-induced SVT may generate a localized form of portal hypertension and then splenoportal or gastroepiploic systems burden following localized venous hypertension and lead to gastric, oesophageal, or colonic varices. It may also cause the liver failure, bowel ischemia, and gastrointestinal bleeding [2]. Patients suffer from great pain and potentially lethal threaten. The limited literature cannot acquire an exact prevalence of SVT in pancreatitis. The aim of the present systematic review and meta-analysis is to obtain the prevalence of SVT in pancreatitis by resolving the following three questions. (1) What is the prevalence of SVT in pancreatitis, including PVT, SplVT, and MVT? (2) What is the prevalence of SVT in different types of pancreatitis? (3) What is the prevalence of SVT in pancreatitis in different regions? This study is conducted according to the guidelines for the reporting of meta-analysis of observational studies, which were published by the Meta-analysis of Observational Studies in Epidemiology Group in 2000 [3].

## 2. Methods

As a systematic review and meta-analysis were planned, we reviewed standard guidelines to conduct meta-analysis studies according to a protocol determined before the study, including study objectives, prespecified eligibility criteria, and methods of statistical analysis.

### 2.1. Eligibility Criteria

1. The participants of any age were diagnosed with pancreatitis, including acute pancreatitis (AP), chronic pancreatitis (CP), hereditary pancreatitis (HP), or autoimmune pancreatitis (AIP). The participants with underlying malignancy, cirrhosis, trauma, abdominal surgery unrelated to ongoing pancreatitis, pregnancy, intra-abdominal infections, primary myeloproliferative disorders, or other pancreatic diseases or pancreatitis and resultant SVT were not deliberately excluded.
2. All cohort and case-control studies were eligible, regardless of the retrospective or prospective nature of the study; case reports were excluded.
3. Reviews, comments, or letters on the relationship of pancreatitis and SVT were excluded.
4. Animal studies were also excluded.
5. There was no publication date or publication status restrictions.
6. There were no language restrictions.
7. The number of participants in any included study was beyond 10.

### 2.2. Search Strategy

The MEDLINE and EMBASE databases were searched using a search strategy from their inception to July 2014. Search items combined keywords and medical subject heading terms (MeSH) were listed as follows: (“pancreatitis” (MeSH Terms) or “pancreatitis” (All Fields)) and ((“portal vein” (MeSH Terms) or (“portal” (All Fields) and “vein” (All Fields)) or “portal vein” (All Fields)) and (“thrombosis” (MeSH Terms) or “thrombosis” (All Fields))) or ((“splenic vein” (MeSH Terms) or (“splenic” (All Fields) and “vein” (All Fields)) or “splenic vein” (All Fields)) and (“thrombosis” (MeSH Terms) or “thrombosis” (All Fields))) or ((“mesenteric veins” (MeSH Terms) or (“mesenteric” (All Fields) and “veins” (All Fields)) or “mesenteric veins” (All Fields) or (“mesenteric” (All Fields) and “vein” (All Fields)) or “mesenteric vein” (All Fields)) and (“thrombosis” (MeSH Terms) or “thrombosis” (All Fields))). The last search was performed on July 29, 2014. The reference lists of the included articles were further hand-searched to identify any additional relevant studies. When the same data were found in more than one publication, only the studies with more complete data and more extensive interval of enrolment were included in the meta-analysis. Full-texts were found by three investigators (Wenda Xu, Xingshun Qi, and Chunping Su).

### 2.3. Data Extraction

Using a predefined protocol, two investigators (Wenda Xu and Xingshun Qi) independently reviewed the titles and abstracts of all references to identify studies for inclusion in the analysis. Dealing with disagreement between the two reviewers, a consensus was achieved through discussion among all of the reviewers. A schematic diagram depicting reference flow is shown through the systematic review process. Additionally, a data extraction sheet was generated that included authors, publication year, study design, country where the study was conducted, period of enrolment, inclusion and exclusion criteria, type of diseases (AP, CP, HP, or AIP), total sample size, demographic data (age and gender), number of patients with PVT, SplV, or MVT, and proportion of patients with PVT, SplV, or MVT, respectively.

### 2.4. Evaluation of Study Quality

Quality assessment of studies was carried out independently by two reviewers (Wenda Xu and Xingshun Qi). Discrepancies of interpretation and comprehension were resolved by consensus. The higher quality studies should fulfill the following predetermined criteria.

1. Country where the study was conducted, interval of enrolment, inclusion and exclusion criteria, and participant characteristics (age, gender) were clearly recorded.
2. Pancreatitis was diagnosed on the basis of history, clinical manifestations, elevated serum lipase and amylase, imaging detection by ultrasonography (US), endoscopic ultrasonography (EUS), computed tomography (CT), magnetic resonance imaging (MRI), or endoscopic retrograde cholangiopancreatography (ERCP), and/or typical histopathology.

### 2.5. Data Synthesis and Statistical Analysis

The proportion of pancreatitis patients with SVT in each study was combined to give a pooled prevalence of SVT for all studies. With this method, the pooled prevalence of PVT, SplV, and MVT was calculated. Furthermore, according to the type of pancreatitis and continents (Europe, America, and Asia), the pooled prevalence of SVT, PVT, SplV, and MVT was also created. The number and crude proportion of participants with SVT recorded by each study were used to pool the overall proportion, using the DerSimonian-Laird random-effects method. Between-study heterogeneity was assessed by using the *I* ^2^ index (*I* ^2^ > 50% was considered having substantial heterogeneity) and the Chi-squared test (*P* < 0.01 was considered representing significant statistical heterogeneity) [4]. Individualized random effects meta-analyses were performed to estimate percentages and 95% confidence intervals (CIs) for all endpoints queried. Analyses were conducted using StatsDirect statistical software version 2.7.8 (StatsDirect Ltd, Sale, Cheshire, UK).

## 3. Results

### 3.1. Description of the Included Studies

**Figure 1:**
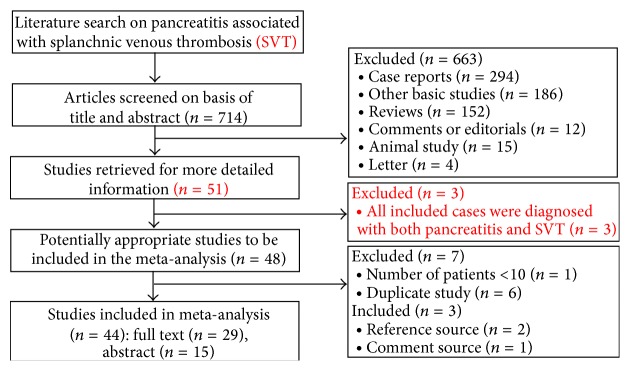
Flow diagram of study selection.

In the initial search strategy, a total of 947 studies were selected. Among them, 714 studies were retrieved by removing duplicate research. Two additional unique references were found through reference lists of an original article and a review, respectively [5, 6]. One study as object of related comment was also identified [7]. Total of 44 studies were included in the meta-analysis (Figure 1). Additionally, five studies recorded by the same study team in different publications were excluded [8–12] and one study which concerned the same patients by the same first author in different publications was also excluded [13]. Another study was excluded because the number of participants was <10 [14]. Twenty-two of 44 studies were conducted in Europe [6, 15–35], fourteen in America [2, 5, 7, 36–46], and eight in Asia [47–54]. Among 10441 participants, 874 patients with SVT were screened. These patients included 197 patients with PVT, 525 with SplV, and 72 with MVT. The detailed characteristics of these included studies were shown in Table 1.

### 3.2. Quality of the Included Studies

The involved countries of all studies could be found. Interval of enrolment was unavailable in ten of the 44 studies. Twenty-one studies had no eligibility criteria; eight of the remaining 23 studies had detailed inclusion and exclusion criteria. Demographic data were completely recorded in nineteen studies. The pancreatitis diagnostic criteria were not elaborate in nine studies only with abstracts and one study with full-test.

### 3.3. Prevalence of SVT in Pancreatitis

**Figure 2:**
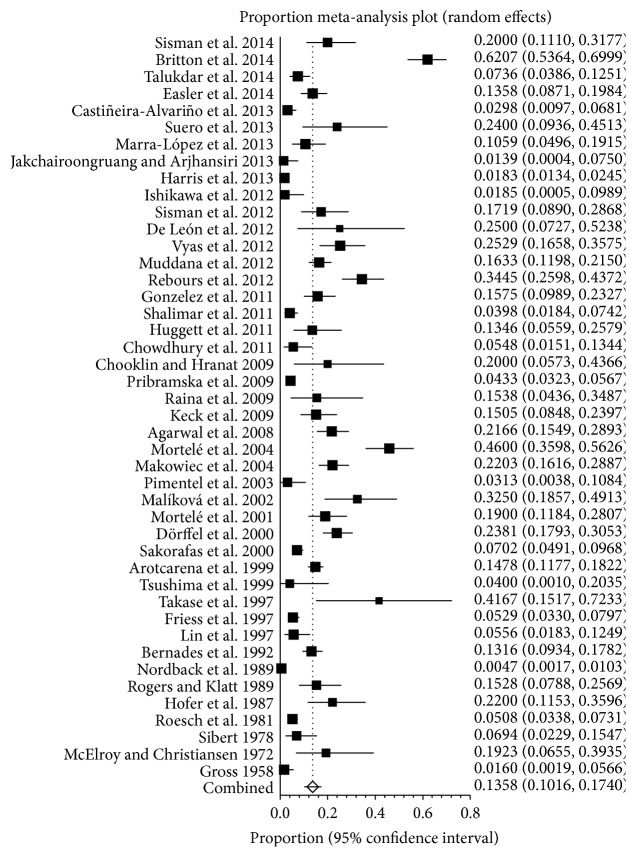
Forest plots showing the prevalence of splanchnic vein thrombosis (SVT) in pancreatitis.

A meta-analysis of involved studies meeting eligibility criteria showed the prevalence of SVT in patients with pancreatitis, ranging from 0.5% to 62.1% (Figure 2). A pooled prevalence was 13.6% (95% CI: 10.2%–17.4%) with a statistically significant heterogeneity among studies (*I* ^2^ = 96.2%, 95% CI: 95.7%–96.6%, *P* < 0.001).

### 3.4. Prevalence of PVT, SplVT, and MVT in Pancreatitis

**Figure 3:**
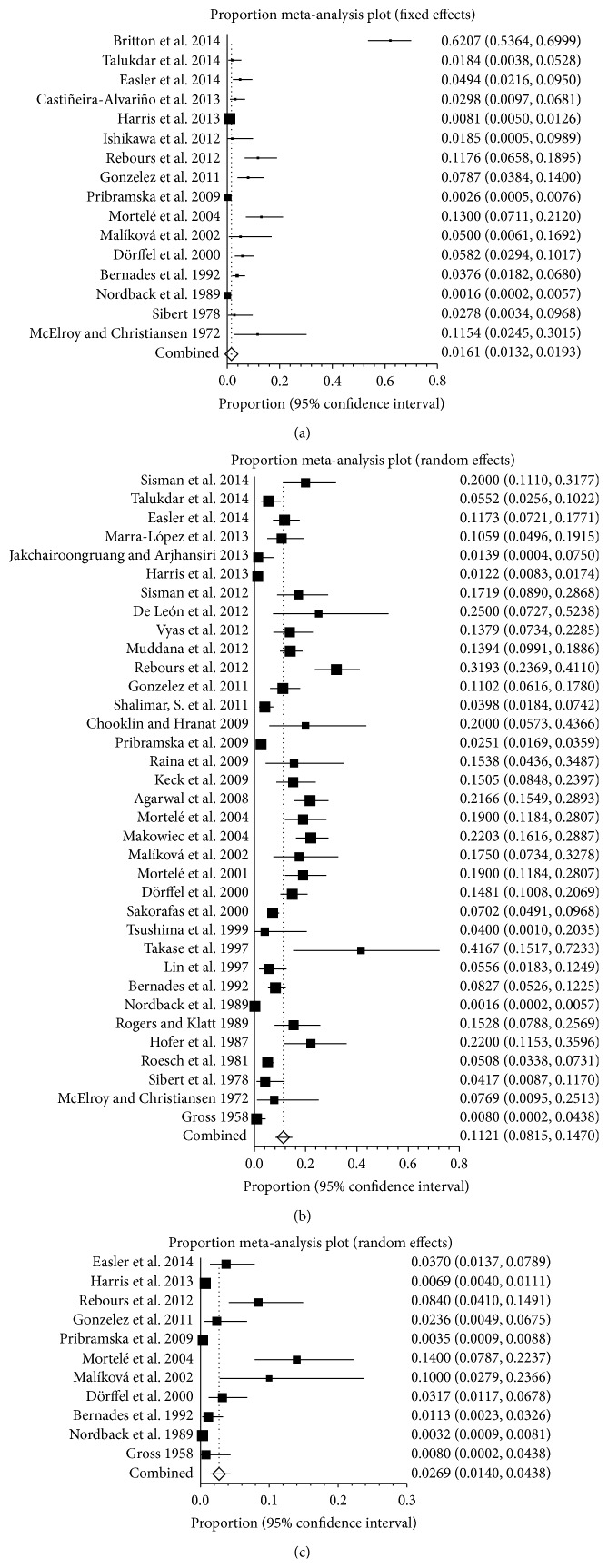
(a) Forest plots showing the prevalence of portal vein thrombosis (PVT) in pancreatitis. (b) Forest plots showing the prevalence of splenic vein thrombosis (SlpVT) in pancreatitis. (c) Forest plots showing the prevalence of mesenteric vein thrombosis (MVT) in pancreatitis.

Seventeen studies reported the prevalence of PVT in patients with pancreatitis, ranging from 0.2% to 62.1% (Figure 3(a)). A pooled prevalence was 6.2% (95% CI: 32.9%–10.7%) with a statistically significant heterogeneity among studies (*I* ^2^ = 97.1%, 95% CI: 96.6%–97.6%, *P* < 0.001). The analysis of patients with SplVT in pancreatitis showed that the prevalence ranged from 0.2% to 41.7% (Figure 3(b)). A pooled prevalence was 11.2% (95% CI: 8.1%–14.7%) with a statistically significant heterogeneity among studies (*I* ^2^ = 95.2%, 95% CI: 94.4%–95.8%, *P* < 0.001). Eleven studies were selected to analyze the prevalence of MVT in pancreatitis, ranging from 0.3% to 14% (Figure 3(c)). The pooled prevalence was 2.7% (95% CI: 1.4%–4.4%) and the heterogeneity remained (*I* ^2^ = 89.3%, 95% CI: 83.2%–92.5%).

### 3.5. Prevalence of SVT in AP and CP

**Figure 4:**
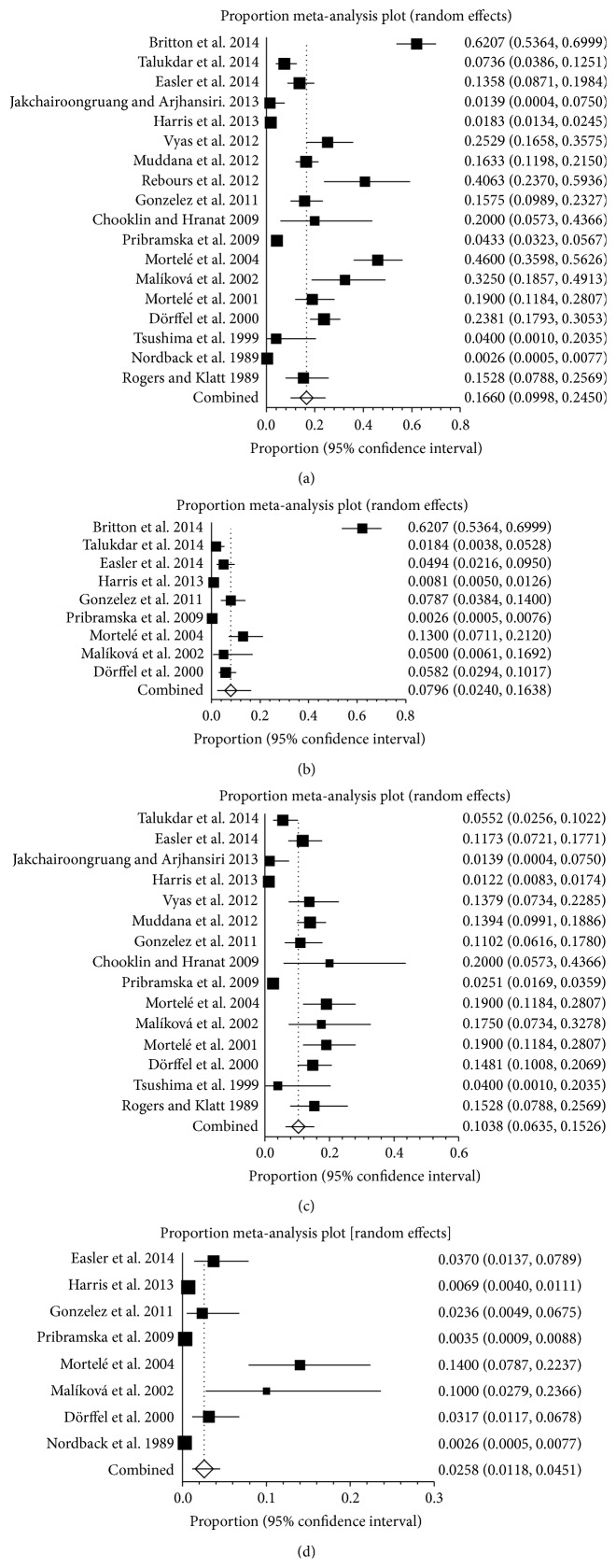
(a) Forest plots showing the prevalence of splanchnic vein thrombosis (SVT) in acute pancreatitis (AP). (b) Forest plots showing the prevalence of portal vein thrombosis (PVT) in acute pancreatitis (AP). (c) Forest plots showing the prevalence of splenic vein thrombosis (SlpVT) in acute pancreatitis (AP). (d) Forest plots showing the prevalence of mesenteric vein thrombosis (MVT) in acute pancreatitis (AP).

Eighteen studies reported the prevalence of SVT in AP, ranging from 0.3% to 62.1% (Figure 4(a)). The pooled prevalence was 16.6% (95% CI: 10.0%–24.5%) with a statistically significant heterogeneity among studies (*I* ^2^ = 98%, 95% CI: 97.7%–98.2%, *P* < 0.001). Nine studies reported the prevalence of PVT in AP, ranging from 0.3% to 62.1% (Figure 4(b)). The pooled prevalence was 8.0% (95% CI: 2.4%–16.4%) with a statistically significant heterogeneity among studies (*I* ^2^ = 98.2%, 95% CI: 97.8%–98.5%, *P* < 0.001). Fifteen studies reported the prevalence of SplVT in AP, ranging from 1.2% to 20% (Figure 4(c)). The pooled prevalence was 10.4% (95% CI: 6.3%–15.3%) with a statistically significant heterogeneity among studies (*I* ^2^ = 94.8%, 95% CI: 93.3%–95.8%, *P* < 0.001). Eight studies reported the prevalence of MVT in pancreatitis, ranging from 0.3% to 14.0% (Figure 4(d)). The pooled prevalence was 2.6% (95% CI: 1.2%–4.5%) with a statistically significant heterogeneity among studies (*I* ^2^ = 90.4%, 95% CI: 83.9%–93.6%, *P* < 0.001).

**Figure 5:**
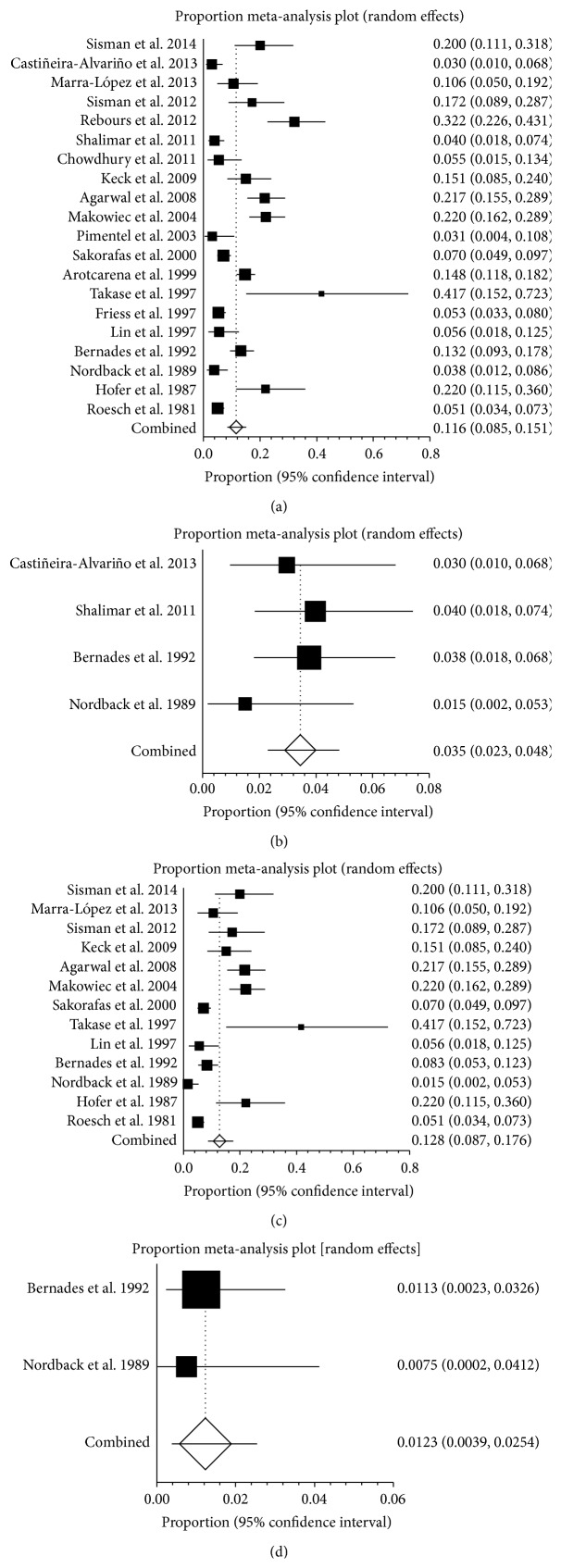
(a) Forest plots showing the prevalence of splanchnic vein thrombosis (SVT) in chronic pancreatitis (CP). (b) Forest plots showing the prevalence of portal vein thrombosis (PVT) in chronic pancreatitis (CP). (c) Forest plots showing the prevalence of splenic vein thrombosis (SlpVT) in chronic pancreatitis (CP). (d) Forest plots showing the prevalence of mesenteric vein thrombosis (MVT) in chronic pancreatitis (CP).

Twenty studies reported the prevalence of SVT in CP, ranging from 3% to 41.7% (Figure 5(a)). The pooled prevalence was 11.6% (95% CI: 8.5%–15.1%) with a statistically significant heterogeneity among studies (*I* ^2^ = 89.5%, 95% CI: 85.6%–91.9%, *P* < 0.001). Four studies reported the prevalence of PVT in CP, ranging from 1.5% to 4% (Figure 5(b)). The pooled prevalence was 3.5% (95% CI: 2.3%–4.8%) and there was no statistical heterogeneity between the two studies (*I* ^2^ = 0%, 95% CI: 0%–67.9%, *P* = 0.5947). Thirteen studies reported the prevalence of SplVT in CP, ranging from 1.5% to 41.7% (Figure 5(c)). The pooled prevalence was 12.8% (95% CI: 8.7%–17.6%) with a statistically significant heterogeneity among studies (*I* ^2^ = 88.8%, 95% CI: 83%–91.9%, *P* < 0.001). Two studies reported the prevalence of MVT in pancreatitis, ranging from 0.8% to 1.1% (Figure 5(d)). The pooled prevalence was 1.2% (95% CI: 0.4%–2.5%). There was no statistical heterogeneity between the two studies (*P* = 0.8506).

### 3.6. Prevalence of SVT in HP and AIP

**Figure 6:**
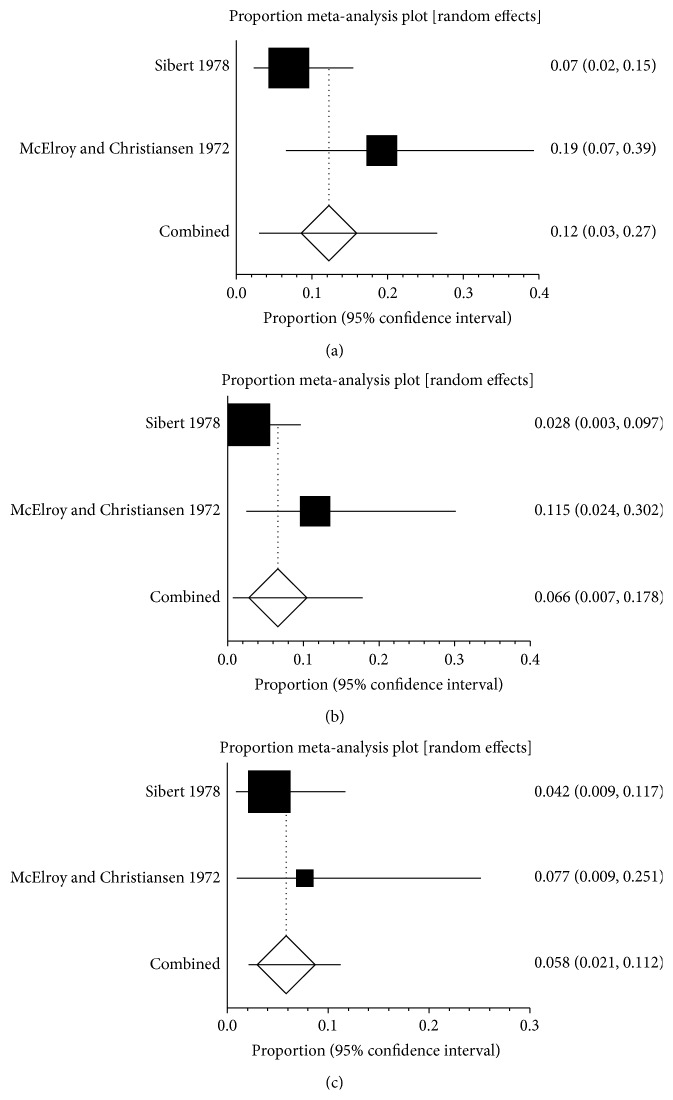
(a) Forest plots showing the prevalence of splanchnic vein thrombosis (SVT) in hereditary pancreatitis (HP). (b) Forest plots showing the prevalence of portal vein thrombosis (PVT) in hereditary pancreatitis (HP). (c) Forest plots showing the prevalence of splenic vein thrombosis (SlpVT) in hereditary pancreatitis (HP).

Only two studies reported the prevalence of SVT in HP, ranging from 7% to 19% (Figure 6(a)). The pooled prevalence was 12.2% (95% CI: 3.0%–26.5%) and there was no statistical heterogeneity between the two studies (*P* = 0.0914). Two studies reported the prevalence of PVT in HP, ranging from 2.8% to 11.5% (Figure 6(b)). The pooled prevalence was 6.6% (95% CI: 0.7%–17.8%) and there was no statistical heterogeneity between the two studies (*P* = 0.1056). Two studies reported the prevalence of SplVT in HP, ranging from 4.2% to 7.7% (Figure 6(c)). The pooled prevalence was 5.8% (95% CI: 2.1%–11.2%) and there was no statistical heterogeneity between the two studies (*P* = 0.437). There was no data on the prevalence of MVT in HP.

**Figure 7:**
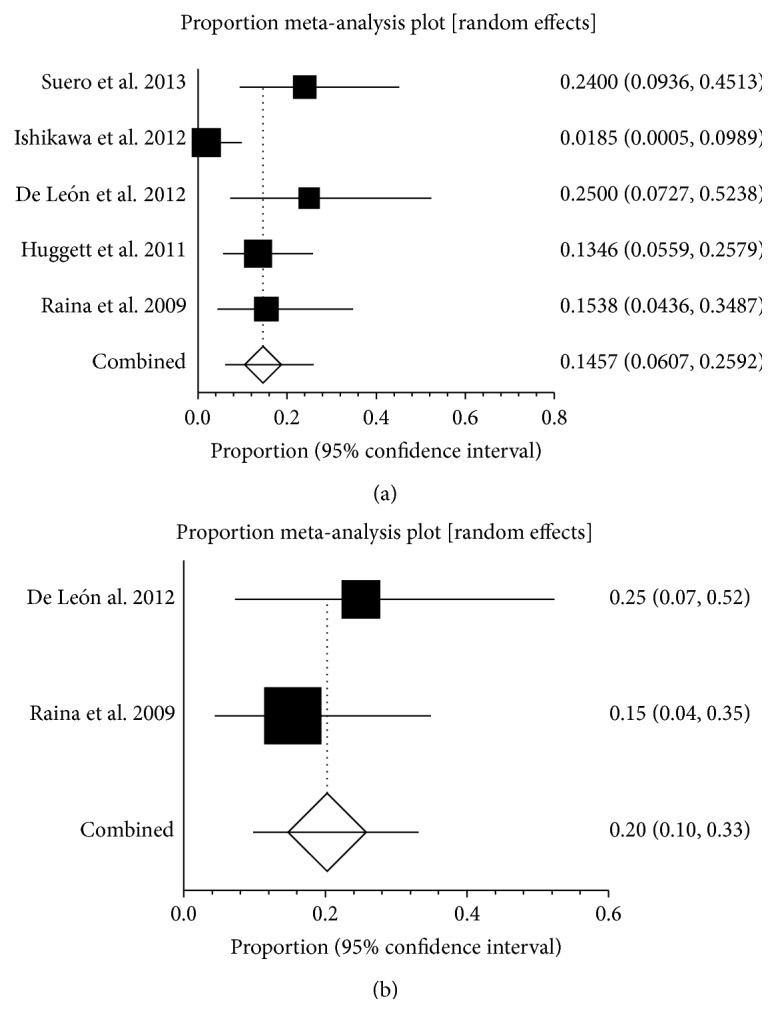
(a) Forest plots showing the prevalence of splanchnic vein thrombosis (SVT) in autoimmune pancreatitis (AIP). (b) Forest plots showing the prevalence of splenic vein thrombosis (SlpVT) in autoimmune pancreatitis (AIP).

Five studies reported the prevalence of SVT in AIP, ranging from 1.9% to 25% (Figure 7(a)). The pooled prevalence was 14.6% (95% CI: 6.1%–25.9%) and there was no statistical heterogeneity between the two studies (*I* ^2^ = 71.4%, 95% CI: 0%–86.7%, *P* = 0.0074). Two studies reported the prevalence of SplVT in AIP, ranging from 15% to 25% (Figure 7(b)). The pooled prevalence was 20.2% (95% CI: 9.8%–33.2%) and there was no statistical heterogeneity between the two studies (*P* = 0.4388). Only one study reported that the prevalence of PVT in AIP was 1.9%. There was no data on the prevalence of MVT in AIP.

### 3.7. Prevalence of SVT in Pancreatitis in Different Continent

**Figure 8:**
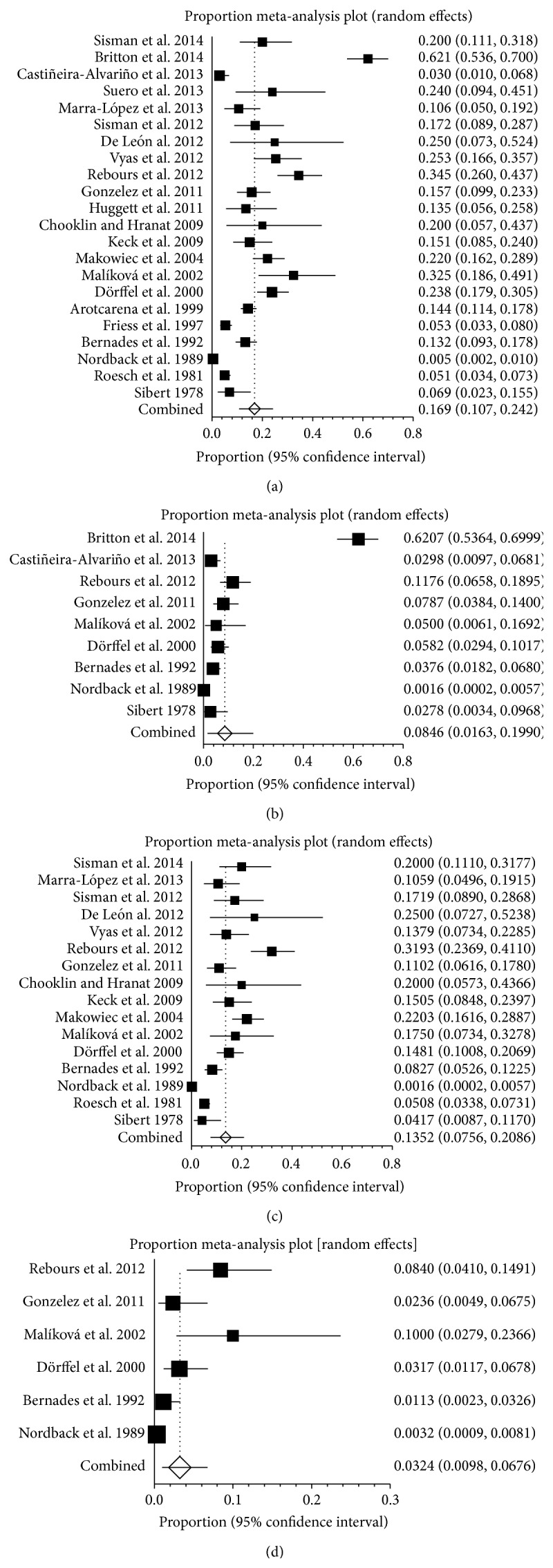
(a) Forest plots showing the prevalence of splanchnic vein thrombosis (SVT) in Europe. (b) Forest plots showing the prevalence of portal vein thrombosis (PVT) in Europe. (c) Forest plots showing the prevalence of splenic vein thrombosis (SlpVT) in Europe. (d) Forest plots showing the prevalence of mesenteric vein thrombosis (MVT) in Europe.

The involved countries of all studies could be divided into three continents (Europe, America, and Asia). The incidences of SVT in pancreatitis were different among three continents. In Europe, twenty-two studies reported the prevalence of pancreatitis patients with SVT, ranging from 0.5% to 62.1% (Figure 8(a)). The pooled prevalence was 16.9% (95% CI: 10.7%–24.2%) with a statistically significant heterogeneity among studies (*I* ^2^ = 97%, 95% CI: 96.6%–97.4%, *P* < 0.001). Nine studies reported the prevalence of PVT in pancreatitis, ranging from 0.2% to 62.1% (Figure 8(b)). The pooled prevalence was 8.5% (95% CI: 1.6%–19.9%) with a statistically significant heterogeneity among studies (*I* ^2^ = 98.1%, 95% CI: 97.7%–98.4%). Sixteen studies reported the prevalence of SplVT in pancreatitis, ranging from 0.2% to 31.9% (Figure 8(c)). The pooled prevalence was 13.5% (95% CI: 7.6%–20.9%) with a statistically significant heterogeneity among studies (*I* ^2^ = 96.2%, 95% CI: 95.3%–96.8%, *P* < 0.001). Six studies reported the prevalence of MVT in pancreatitis, ranging from 0.3% to 10% (Figure 8(d)). The pooled prevalence was 3.2% (95% CI: 1.0%–6.8%) with a statistically significant heterogeneity among studies (*I* ^2^ = 88.8%, 95% CI: 77.4%–93.1%, *P* < 0.001).

**Figure 9:**
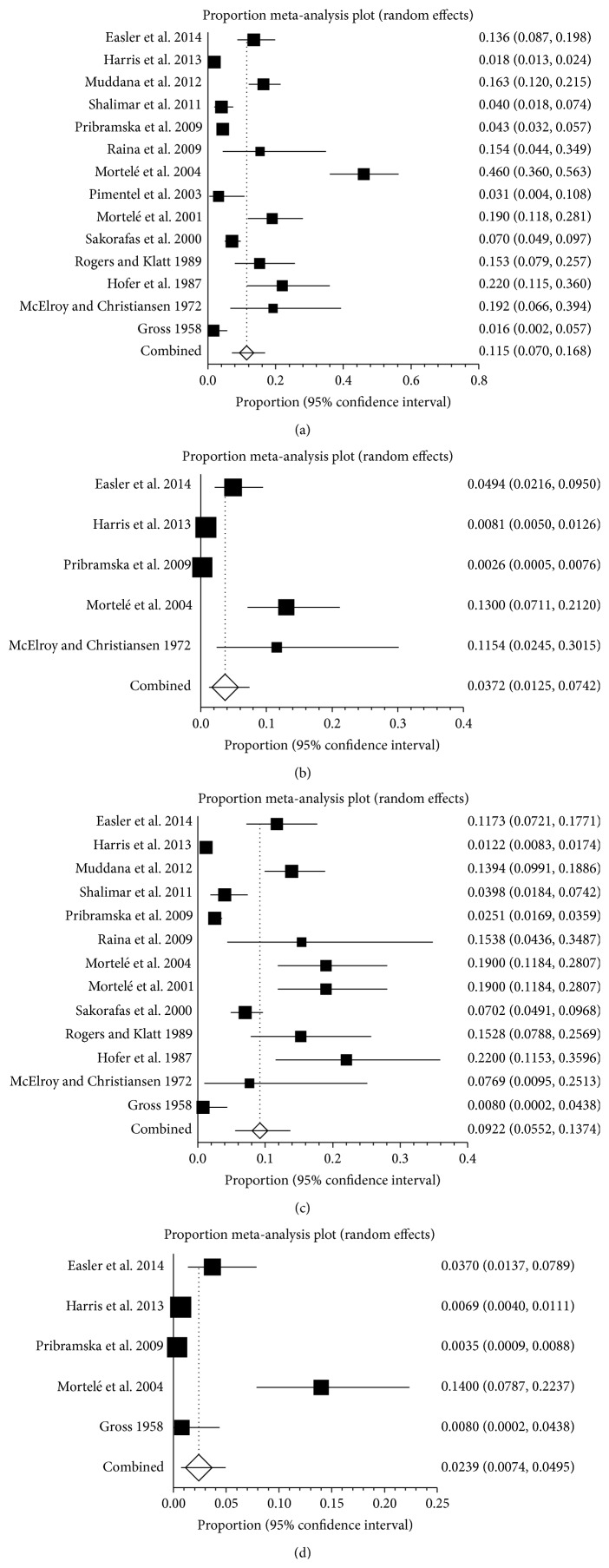
(a) Forest plots showing the prevalence of splanchnic vein thrombosis (SVT) in America. (b) Forest plots showing the prevalence of portal vein thrombosis (PVT) in America. (c) Forest plots showing the prevalence of splenic vein thrombosis (SlpVT) in America. (d) Forest plots showing the prevalence of mesenteric vein thrombosis (MVT) in America.

In America, fourteen studies reported the prevalence of SVT in pancreatitis, ranging from 1.6% to 46% (Figure 9(a)). The pooled prevalence was 11.5% (95% CI: 7.0%–16.8%) with a statistically significant heterogeneity among studies (*I* ^2^ = 95.7%, 95% CI: 94.6%–96.5%, *P* < 0.001). Five studies reported the prevalence of PVT in pancreatitis, ranging from 0.3% to 13% (Figure 9(b)). The pooled prevalence was 3.7% (95% CI: 1.3%–7.4%) with a statistically significant heterogeneity among studies (*I* ^2^ = 93.2%, 95% CI: 87.6%–95.6%, *P* < 0.001). Thirteen studies reported the prevalence of SplVT in pancreatitis, ranging from 0.8% to 22% (Figure 9(c)). The pooled prevalence was 9.2% (95% CI: 5.5%–13.7%) with a statistically significant heterogeneity among studies (*I* ^2^ = 94.8%, 95% CI: 93.2%–95.9%, *P* < 0.001). Five studies reported the prevalence of MVT in pancreatitis, ranging from 0.4% to 14% (Figure 9(d)). The pooled prevalence was 2.4% (95% CI: 0.7%–5.0%) with a statistically significant heterogeneity among studies (*I* ^2^ = 91.7%, 95% CI: 83.6%–94.8%, *P* < 0.001).

**Figure 10:**
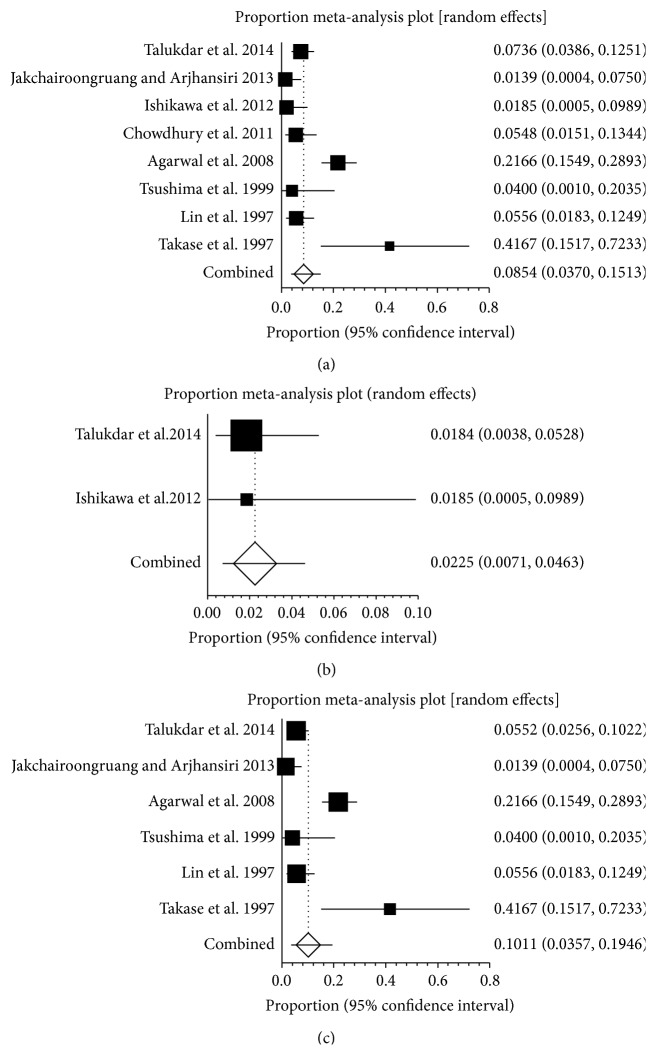
(a) Forest plots showing the prevalence of splanchnic vein thrombosis (SVT) in Asia. (b) Forest plots showing the prevalence of portal vein thrombosis (PVT) in Asia. (c) Forest plots showing the prevalence of splenic vein thrombosis (SlpVT) in Asia.

In Asia, eight studies reported the prevalence of SVT in pancreatitis, ranging from 1.4% to 41.7% (Figure 10(a)). The pooled prevalence was 8.5% (95% CI: 3.7%–15.1%) with a statistically significant heterogeneity among studies (*I* ^2^ = 84.2%, 95% CI: 68.5%–90.3%, *P* < 0.001). Two studies reported the prevalence of PVT in pancreatitis, ranging from 1.8% to 1.9% (Figure 10(b)). The pooled prevalence in two studies was 2.3% (95% CI: 0.7%–4.6%). Six studies reported the prevalence of SplVT in pancreatitis, ranging from 1.4% to 41.7% (Figure 10(c)). The pooled prevalence was 10.1% (95% CI: 3.6%–19.5%) with a statistically significant heterogeneity among studies (*I* ^2^ = 87.8%, 95% CI: 74.5%–92.6%, *P* < 0.001). There was no data on the prevalence of MVT in pancreatitis in Asia.

## 4. Discussion

The previous published meta-analysis that assessed the prevalence of SVT in patients with pancreatitis almost focused on AP, CP, HP, or AIP alone without systematic search. The prevalence varied from 1% to 24% in previous studies according to the type of pancreatitis and imaging technique used (US, EUS, CT, MRI, or ERCP) [30, 40, 55]. However, our study was remarkably different from the previous ones, as follows. (1) In the previous studies, most of them were single-center studies with a limited enrollment period and target population. By comparison, our meta-analysis included all studies conducted from 1958 to 2014 and 10560 patients with pancreatitis. Furthermore, we made a distinction on the source of patients according to the continents. (2) As we have known, SVT involves PVT, SplVT, and MVT. In contrast to the previous studies, we have paid more attention on the prevalence of PVT, SplVT, and MVT in pancreatitis. (3) Our research reported the prevalence of SVT on the basis of the different types of pancreatitis including AP, CP, HP, and AIP. However, there was no similar record in the previous studies.

Pancreatitis is associated with a variety of vascular complications including SVT. Clinically, SVT in pancreatitis is becoming common with the advancement of imaging technique. In the current systematic review and meta-analysis, we demonstrated that 13.6% of pancreatitis had SVT, 6.2% had PVT, 11.2% had SplVT, and 2.7% had MVT. The prevalence of SVT in pancreatitis showed some regional differences. We found that the prevalence of SVT in pancreatitis in Europe reached 16.9%, which was the highest among the three continents. Moreover, the prevalence of PVT, SplVT, and MVT in pancreatitis in Europe was higher than that in America or in Asia, respectively. We can find from the involved studies that the results came from nine countries of Europe, which were more than those from four of Asia and two of America. Compared with Asia, the prevalence of SVT in pancreatitis was 11.5% higher than that of 8.5% in Asia, but the prevalence of SplVT was a little lower in America.

Previous studies that reported the vascular complications of pancreatitis showed different prevalence of SVT in pancreatitis regardless of the type of pancreatitis. This meta-analysis demonstrated that the prevalence of SVT in AP was 16.6% higher than previous studies with a reported incidence of 1-2% [1]. The reason was that previous studies took PVT, SplVT, and MVT inclusion of SVT as numerator. The controversy also exists about the prevalence of SplVT in CP. The old series reported that the prevalence of SplVT in patients with CP varied between 2.5% and 25% [6, 51, 56, 57]. Our results showed 12.8% CP with SplVT, which was similar to that from a recent report by Butler et al. [58]. However, there was no detailed description on the prevalence of SVT in HP and AIP. Our results showed that the prevalence of SVT was 12.2% and 14.6% in HP and AIP, respectively. All of these could enrich the whole research on the prevalence of SVT in pancreatitis.

## 5. Limitation

Our study has several limitations. Firstly, the heterogeneity of available data from various years' studies was significant. Depending on advanced imaging technique, the majority of asymptomatic patients with SVT could be involved in research groups. Therefore, only the random-effects model was applied in our meta-analysis to generate a more conservative estimate of the proportion. Secondly, there are different results between Western and Asian countries. Besides the difference in the population race, lifestyle, and diagnostic level, the number of countries involved in the research is different. America included two countries and four countries were from Asia. Thirdly, there is no related research on the prevalence of SVT in HP and AIP. The final results may be not so typical.

## 6. Conclusion

This systematic review and meta-analysis attempt to quantify the incidence of SVT in pancreatitis according to the different forms of pancreatitis and regional distribution. Further studies are needed to analyze the relationship between natural history, clinical significance, long-term outcomes, and the prevalence of SVT in pancreatitis. The rate of SVT associated gastrointestinal bleeding and the security and reasonability of anticoagulation therapy on thrombosis are all needed to develop complete, large, multicentre, and collaborative studies.

## Figures and Tables

**Table 1:** Overview of included studies.

Authors (publication year)	Type of research	Country	Interval of enrolment	Eligibility criteria	Type of disease (AP, CP, HP, or AIP)		Number of total samples	SVT	PVT	SlpVT	MVT	Mean age (range years)	Female/ male
Sisman et al. (2014) [19]	Retrospective * *cohort study	Turkey	2007–2011	NA		CP	65	13	NA	13	NA	NA	14/51

Britton et al. (2014) [18]	Retrospective * *cohort study	UK	1998–2012	Exclusion criteria: 46 cases were excluded from the final analysis as imaging reports made no comment on the portal venous system.	AP		145	90	90	NA	NA	56.1	NA

Talukdar et al. (2014) [47]	Prospective * * cohort study	India	August 2011 to October 2012	Inclusion criteria: patients over 18 yrs with a primary diagnosis of first episode of AP from August 2011 to October 2012, and prospectively followed for at least six months after discharge or till death; exclusion criteria were (1) recurrent AP; (2) patient who did not get a CT scan.	AP		163	12	3	9	NA	NA	40/123

Easler et al. (2014) [38]	Prospective study	USA	June 2003 and April 2010	Inclusion criteria: SAP patients who were admitted with their first AP attack; exclusion criteria: patients with a history of AP or CP.	AP		162	22	8	19	6	55 ± 20	78/84

Castiñeira-Alvariño et al. (2013) [20]	Prospective study	Spain	Since 2007	Inclusion criteria: patients with age <18 years at index visit to the CP outpatient clinic; exclusion criteria: patients who clearly modified the diet before the index visit were excluded.		CP	168	5	5	NA	NA	44 (17–76)	40/128

Suero et al. (2013) [21]	Retrospective study	Spain	NA	NA		AIP	25	6	NA	NA	NA	(17–79)	2/23

Marra-López et al. (2013) [22]	Prospective cohort study	Spain	2011–2013	Inclusion criteria: CP patients defined by M-ANNHEIM.		CP	85	9	NA	9/85	NA	59.38	17/68

Harris et al. (2013) [2]	Retrospective study	USA	January 1996 to December 2006	Inclusion criteria: an institutional (Mayo clinic) database search was done using the key terms acute pancreatitis and superior mesenteric vein thrombosis (SMVT), portal vein thrombosis (PVT), and splenic vein thrombosis (SplVT) from January 1996 to December 2006; exclusion criteria: (1) patients with precipitating factors for thrombosis (abdominal surgery unrelated to ongoing pancreatitis, trauma, pregnancy, cirrhosis, intra-abdominal infections, pancreatic cancer, and primary myeloproliferative disorders) were excluded. (2) All cases of chronic pancreatitis were also excluded.	AP		2454	45	20	30	17	NA	NA

Jakchairoongruang and Arjhansiri (2013) [48]	Retrospective study	Thailand	January 1, 2005 and April 30, 2010	Exclusion criteria: six hundred and eight patients were excluded from the study population because of the following reasons: it was not the first episode of acute pancreatitis, the initial CT was not performed, there are no available CT images on our Pictures Archiving and Communications System (PACS), or patient was imaged with only unenhanced CT.	AP		72	1	NA	1/72	NA	47.7 (6–89)	33/39

Ishikawa et al. (2012) [49]	Retrospective study	Japan	July 2003 and October 2010	Inclusion criteria: patients who met the International Consensus Diagnostic Criteria (ICDC) for AIP.		AIP	54	1	1	NA	NA	63.2 ± 13.5 (28–86)	6/48

Sisman et al. (2012) [24]	Retrospective study	Turkey	2007–2011	Exclusion criteria: patients who received previous H. pylori eradication treatment and who are with any malignancy were excluded.		CP	64	11	NA	11	NA	NA	NA

De León et al. (2012) [25]	Retrospective study	Spain	NA	NA		AIP	16	4	NA	4	NA	46.6 (17–73)	1/15

Vyas et al. (2012) [23]	Retrospective study	UK	NA	NA	AP		87	22	NA	12	NA	NA	35/52

Muddana et al. (2012) [39]	Prospective study	USA	2003–2010	NA	AP		251	41	NA	35	NA	51.5 ± 19	123/128

Rebours et al. (2012) [17]	Prospective single-center study	France	2000–2009	Included criteria: all of the in- or outpatients with recurrent acute or chronic alcoholic pancreatitis and followed prospectively based on a standardized protocol, including a yearly physical examination and a search for exocrine pancreatic insufficiency, diabetes, and cholestasis; excluded criteria: (1) cirrhosis; (2) patients who were lost to follow-up in 2009.	AP or CP		119	41	14	38	10	NA	19/100

Gonzelez et al. (2011) [15]	Retrospective study	UK	January 1, 2008, and December 31, 2009	Excluded criteria: patients with chronic pancreatitis, known malignancy, cirrhosis, or established portal hypertension.	AP		127	20	10	14	3	53.5 (36–81)	118/9

Shalimar et al. (2011) [36]	Prospective study	USA	NA	Inclusion criteria: patients with CP after an informed consent and ethical clearance; exclusion criteria: NA.		CP	226	9	NA	9	NA	NA	48/178

Huggett et al. (2011) [16]	Prospective study	UK	2004–2010	Inclusion criteria: patients with AIP and with a median follow-up from diagnosis of 32 months (range 0–76)		AIP	52	7	NA	NA	NA	59 (26–85)	11/41

Chowdhury et al. (2011) [50]	Retrospective study	India	January 2005 and December 2009	Inclusion criteria: children diagnosed with chronic pancreatitis (on imaging study).		CP	73	4	NA	NA	NA	NA	22/51

Chooklin and Hranat (2009) [26]	Retrospective study	Ukraine	NA	NA	AP		20	4	NA	4	NA	NA	3/17

Vege et al. (2009) [37]	Retrospective study	USA	1989–2007	Exclusion criteria: patients with preexisting portal hypertension were excluded.	AP		1155	50	3	29	4	NA	NA

Keck et al. (2009) [27]	Retrospective study	Germany	In Germany: 2001–2005; in USA: 1995–2005	Excluded criteria: three patients whose predominant lesion was only a very dilated pancreatic duct and who were thus selected for lateral pancreaticojejunostomy.		CP	93	14	NA	14	NA	NA	31/62

Raina et al. (2009) [5]	Retrospective study	USA	1998–2007	Exclusion criteria: three other malignancies (gastric cancer, the recurrence of gastric adenocarcinoma, and pancreatic adenocarcinoma).		AIP	26	4	NA	4	NA	62.5 (23–86)	9/17

Agarwal et al. (2008) [51]	Retrospective study	India	January 1996 and December 2005	NA		CP	157	34	NA	34	NA	NA	NA

Mortelé et al. (2004) [40]	Retrospective study	USA	17-month period	NA	AP		100	46	13	19	14	51 (12–80)	47/53

Makowiec et al. (2004) [28]	Prospective study	Germany	1994–2001	Exclusion criteria: 12 patients with portal hypertension and 9 patients underwent splenectomy without splenic vein thrombosis.		CP	177	NA	NA	39	NA	NA	NA

Pimentel et al. (2003) [41]	Retrospective study	Brazil	1989–2002	NA		CP	64	2	NA	NA	NA	NA	14/50

Malíková et al. (2002) [29]	Retrospective study	Czech Republic	NA	NA	AP		40	NA	2	7	4	NA	NA

Mortelé et al. (2001) [42]	Retrospective study	USA	April 1996 and August 1997	NA	AP		100	19	NA	19	NA	NA	47/53

Sakorafas et al. (2000) [43]	Prospective study	USA	1976–1997	NA		CP	484	34	NA	34	NA	NA	NA

Dörffel et al. (2000) [30]	Prospective study	Germany	38 months	Included criteria: definite diagnosis of acute pancreatitis as detected by ultrasonography (US) and CT, elevated serum lipase and amylase, compatible clinical picture, onset of pain symptoms not more than 3 days before admission, no history of pancreatitis, and no deficiencies in antithrombin III, protein C, and protein S.	AP		189	45	11	28	6	43 (19–80)	49/140

Arotcarena et al. (1999) [31]	Prospective study	France	April 1993 to April 1996	NA		CP	494	73	NA	NA	NA	NA	NA

Tsushima et al. (1999) [52]	Retrospective study	Japan	April 1993 and August 1997	Included criteria: patients with AP underwent initial abdominal CT within 3 days after the onset of symptoms and received at least one additional CT examination thereafter; Excluded criteria: cases of traumatic or postoperative pancreatitis	AP		25	1	NA	1	NA	53.4 ± 20.8 (25–83)	7/18

Takase et al. (1997) [54]	Prospective study	Japan	During the past 10 years	Exclusion criteria: 6 patients underwent pancreatoduodenectomy or biopsy without the splenic vein specimens		CP	12	5	NA	5	NA	48.8	0/12

Friess et al. (1997) [32]	Retrospective study	Greece	NA	NA		CP	397	21	NA	NA	NA	NA	NA

Lin et al. (1997) [53]	Prospective study	Taiwan	1976–1996	NA		CP	90	5	NA	5	NA	NA	NA

Bernades et al. (1992) [6]	Prospective longitudinal Study	France	January 1980 and March 1990	Inclusion criteria: patients who met the criteria of CP and who had been followed-up during observation; exclusion criteria: patients with cirrhosis.		CP	266	35	10	22	3	40.5 (6–77)	35/231

Nordback et al. (1989) [35]	Retrospective study	Finland	1972–1986	NA	AP or CP		1268	6	2	2	4	NA	NA

Rogers and Klatt (1989) [44]	Retrospective autopsy case-control study	USA	January 1958 and December 1987	Inclusion criteria: cases were included for study if acute pancreatitis was the immediate or contributing cause of death and the portal venous system (both splenic and portal veins) was examined completely at autopsy. Exclusion criteria: patients were excluded from study if there had been a splenectomy or if chronic pancreatitis was present microscopically.	AP		72	11	NA	11	NA	NA	20/52

Hofer et al. (1987) [45]	Prospective study	USA	1975–1985	NA		CP	50	11	NA	11	NA	50 (13–75)	24/26

Roesch et al. (1981) [33]	Retrospective study	Germany	NA	NA		CP	531	27	NA	27	NA	42.5	49/482

Sibert (1978) [34]	Retrospective study	UK	NA	NA	HP		72	5	2	3	NA	NA	NA

McElroy and Christiansen (1972) [46]	Retrospective study	USA	NA	NA	HP		26	5	3	2	NA	NA	NA

Gross (1958) [7]	Prospective study	USA	1956	NA	AP or CP		125	2	NA	1	1	49 (6–75)	34/91

NA: the data are not available; AP: acute pancreatitis; CP: chronic pancreatitis; HP: hereditary pancreatitis; AIP: autoimmune pancreatitis; SVT: splanchnic vein thrombosis; PVT: portal vein thrombosis; SlpVT: splenic vein thrombosis; MVT: mesenteric vein thrombosis.
